# Topical Oxygen Therapy and a Zinc-Containing Palatal Stent for Post-surgical Healing: A Case Report of a Large Irritation Fibroma of the Hard Palate

**DOI:** 10.7759/cureus.109714

**Published:** 2026-05-27

**Authors:** Minas Leventis, Ioanna Mitsika

**Affiliations:** 1 Oral and Maxillofacial Surgery, Dental School, National and Kapodistrian University of Athens, Athens, GRC; 2 Dentistry, National and Kapodistrian University of Athens, Athens, GRC

**Keywords:** benign tumor, hard palate, irritation fibroma, oxygen therapy, palatal stent, palatal surgery, reactive hyperplasia, zinc-embedded polymer

## Abstract

Irritation fibroma is a common benign reactive lesion of the oral mucosa, typically resulting from chronic mechanical irritation. While generally small and asymptomatic, rare cases may exhibit atypical growth.

This report presents a 37-year-old male patient with a 2.7 cm fibroma located on the hard palate. Complete surgical excision was performed, and histopathological examination confirmed the diagnosis of irritation fibroma with no evidence of dysplasia or malignancy. Postoperative management included the use of a chairside-fabricated zinc-infused palatal stent, combined with topical oxygen- and lactoferrin-containing oral gel, to support wound protection during secondary healing. The postoperative course was uneventful, with satisfactory mucosal healing and no recurrence observed at one-year follow-up.

This case presents the clinical presentation and management of an unusually large palatal irritation fibroma and highlights a supportive postoperative protocol that may constitute a useful adjunct in oral wound healing.

## Introduction

Irritation fibroma, also referred to as fibrous hyperplasia, is a benign lesion commonly found in the oral cavity. Clinically, it typically presents as an exophytic nodular lesion with a smooth or occasionally inflamed mucosal surface, firm consistency, and a color ranging from pink to that of the adjacent mucosa. Irritation fibromas are usually well-circumscribed, and their size rarely exceeds 1.5 cm in diameter [1-3].

Although generally asymptomatic, these lesions may cause discomfort when located in areas subjected to frequent mechanical irritation, such as the hard palate. Irritation fibromas account for approximately 4.5% of all oral mucosal lesions. The reported prevalence among adults is approximately 1.2%, with a female predominance. These lesions are most commonly observed between the third and sixth decades of life. Although irritation fibromas may occur at any intraoral site, they are most frequently found along the occlusal plane of the buccal mucosa [1-3].

The primary etiology of irritation fibromas is believed to be chronic mechanical trauma caused by factors such as cheek biting, sharp tooth edges, inadequate dental prosthesis margins, abrasive food intake, or poorly fitting dentures. In addition, exposure to local irritants, including tobacco use, has also been implicated as a contributing factor. Clinical examination and patient history are essential, with diagnosis reached through histopathological analysis. Histologically, irritation fibroma is characterized by a proliferation of dense fibrous connective tissue, often associated with hyperplastic or hyperkeratotic changes in the overlying epithelium. The connective tissue typically demonstrates dense collagen fiber bundles and localized hyperplasia of mature fibroblasts [1-3].

The present report describes the management of a large palatal irritation fibroma measuring more than 2 cm in diameter in a male patient. In addition to the clinical and histopathological findings, the report describes the postoperative use of a chairside-fabricated zinc-infused thermoplastic palatal stent combined with topical oxygen- and lactoferrin-releasing oral gel during secondary healing following surgical excision.

## Case presentation

A 37-year-old male patient with no significant medical history presented with a lesion involving the left hard palate that had been present for approximately three years. Two years prior to presentation, an incisional punch biopsy had been performed in a hospital setting. Histopathological findings were consistent with an irritation fibroma, with no evidence of malignancy identified. Despite the benign diagnosis, the patient did not pursue further treatment, and the lesion progressively enlarged over time. The patient reported smoking approximately 20 cigarettes daily and denied alcohol consumption. No obvious local traumatic factors, including sharp tooth edges, defective restorations, prosthetic irritation, or occlusal trauma, were identified during clinical examination. Although the patient was a smoker, no specific smoking-related irritation was detected at the lesion site. There was no history of infection associated with the anterior hard palate region. Initially, the lesion was asymptomatic; however, the patient later reported discomfort during mastication and speech. Intraoral examination revealed a firm, solitary, well-circumscribed nodular lesion measuring approximately 2.7 cm in diameter with a pedunculated base. The surface of the lesion was covered by normal-colored mucosa with a central area of ulceration. No tooth mobility was detected in the adjacent region, and pulp vitality tests of the teeth adjacent to the lesion were positive (Figure 1).

**Figure 1:**
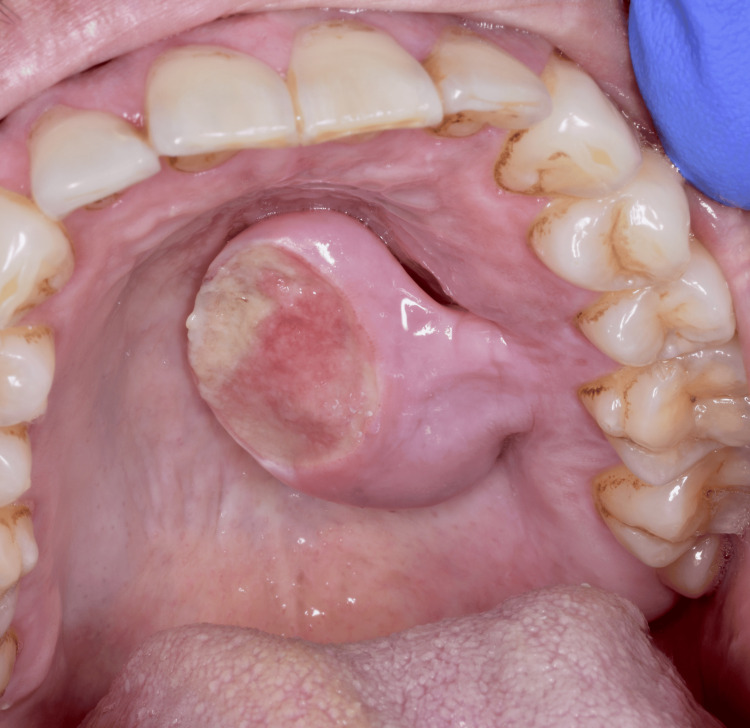
Initial clinical view of an erythematous palatal lesion with a central ulcerated surface.

A cone-beam computed tomography (CBCT) scan of the maxilla and paranasal sinuses was performed to evaluate the extent of the palatal lesion and to assess possible involvement of adjacent osseous structures due to the lesion’s unusually large size and prolonged clinical duration. Imaging revealed a well-defined, homogeneous soft-tissue mass involving the left hard palate without evidence of osseous involvement. Adjacent anatomical structures, including the alveolar process, palatal cortical plate, nasal floor, and maxillary sinus walls, remained intact, with no evidence of bone destruction or additional hard tissue pathology. Mild physiological pneumatization, with slight Schneiderian membrane thickening at the floor of the left maxillary sinus, was noted. No odontogenic or endodontic pathology was identified (Figure 2).

**Figure 2:**
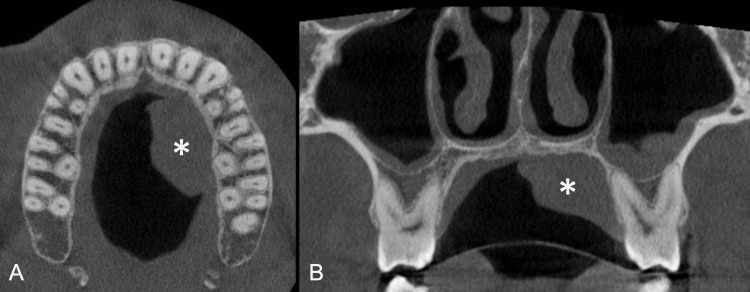
Axial (A) and coronal (B) views of the CBCT showing the soft tissue mass (asterisk), revealing no additional hard or soft tissue pathology. CBCT: cone-beam computed tomography

The patient underwent surgical excision of the lesion under local anesthesia. The excised specimen was immediately fixed in neutral buffered formalin and submitted for histological analysis. Following surgery, Blue®m oral gel (blue®m, Zwolle, Netherlands) was applied to the surgical site. A palatal stent was fabricated chairside using a novel zinc-infused thermoplastic polymer (Oral Surgical Granulate®, Elemental, Belgium) to protect the palatal wound immediately after surgery and throughout the initial healing period. The fabrication protocol and clinical application of this chairside-fabricated palatal stent have been described in detail in a recent case report [4]. The patient was instructed on the proper insertion, removal, and cleaning of the palatal surgical stent and was advised to wear it for seven days, maintaining it in place for as many hours per day as possible. In addition, the patient was instructed to apply a small amount of Blue®m oral gel to the internal surface of the Elemental palatal stent three times daily before placement (Figure 3).

**Figure 3:**
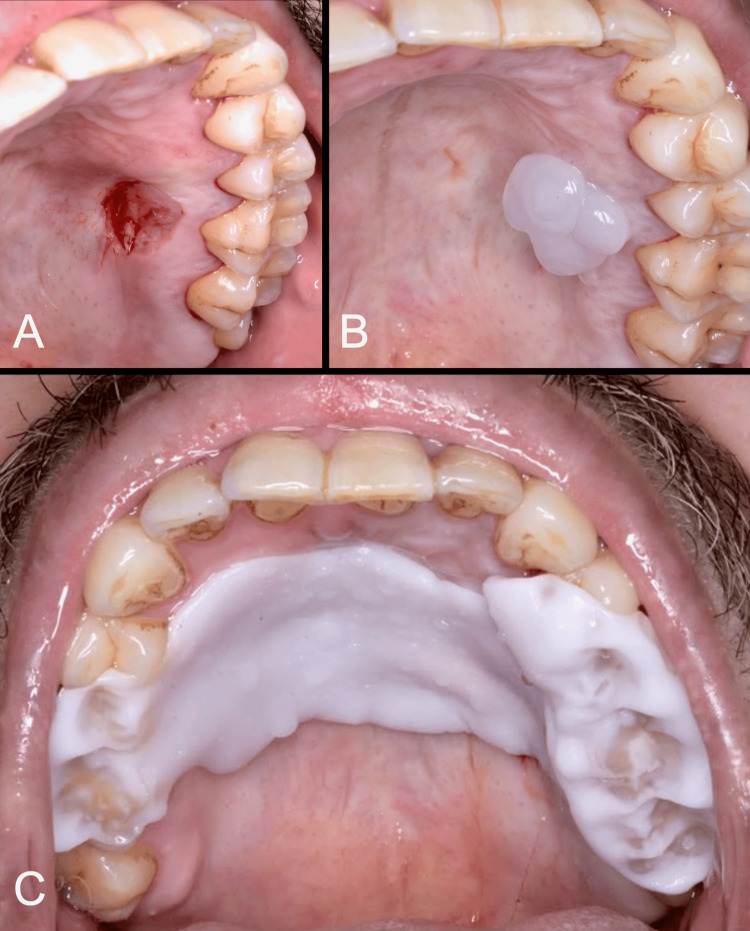
Management included excisional biopsy (A), adjunctive topical oxygen and lactoferrin therapy (B), and placement of a zinc-containing palatal stent (C).

The patient was given postoperative instructions and prescribed ibuprofen 400 mg to be taken every eight hours. Additionally, Blue®m oral gel was provided, with directions to apply it to the inner surface of the Elemental palatal stent three times daily, ensuring sufficient contact with the healing tissues to support recovery. At the one-year follow-up evaluation, the patient remained asymptomatic, with complete mucosal healing and no signs of lesion recurrence (Figure 4).

**Figure 4:**
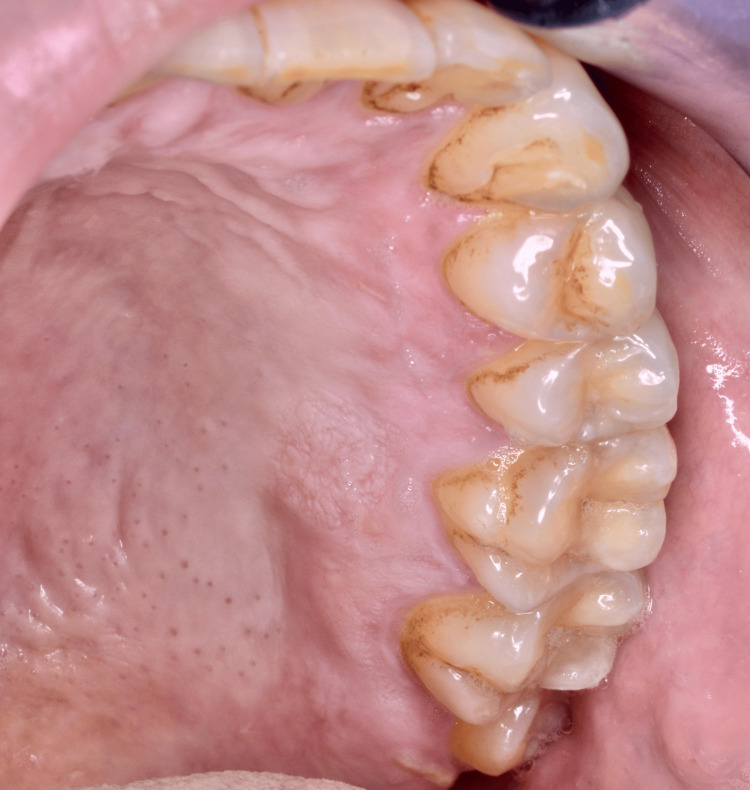
At the one-year follow-up, no evidence of recurrence was observed.

The histological specimen consisted of a whitish, firm nodular lesion measuring 2.7 cm in maximum diameter, with a partially ulcerated surface measuring approximately 2 cm. Microscopically, the lesion consisted of a protruding, partially ulcerated, non-encapsulated nodule composed of fibrous connective tissue with dense collagen bundles, fibroblasts, blood vessels, and inflammatory cell infiltration predominantly located beneath the ulcerated surface. The overlying stratified squamous epithelium demonstrated no atypical features. Complete excision of the irritation fibroma was confirmed, with no evidence of cellular atypia or malignancy identified (Figure 5).

**Figure 5:**
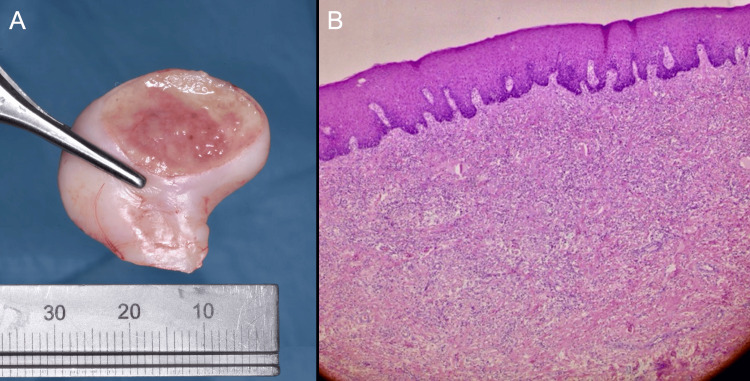
Complete excision of the lesion (A) and histological image consistent with an irritation fibroma (B).

## Discussion

Irritation fibroma, also known as traumatic fibroma, is one of the most common benign lesions of the oral mucosa. Its development is typically associated with chronic irritation or trauma and commonly affects areas such as the buccal mucosa, gingiva, and tongue. The primary etiological factor is prolonged mechanical trauma, including irritation caused by ill-fitting dentures or habitual cheek biting, as supported by the findings of Lapitskaya et al. (2022) [5]. Furthermore, environmental and cultural habits, including tobacco or betel nut chewing, have also been associated with an increased risk of irritation fibroma development, highlighting the multifactorial nature of this lesion [1]. In the present case, no definitive traumatic source was identified clinically. However, chronic low-grade mechanical irritation related to tongue contact with the lesion was considered a possible contributing factor to its gradual enlargement over time.

According to the literature, irritation fibromas are generally asymptomatic, slow-growing lesions that rarely exceed 1.5 cm in diameter. Clinically, they commonly present as exophytic nodules with a smooth surface and firm consistency. In the present case, however, the lesion measured approximately 2.7 cm in diameter, which is considerably larger than typically reported. It is possible that the lesion initially developed as a small fibroma and gradually enlarged over time due to continuous low-grade irritation and the anatomical capacity of the hard palate to accommodate progressive growth without causing significant symptoms.

Histopathological examination typically demonstrates dense collagenized connective tissue with minimal vascularity and the presence of chronic inflammatory cell infiltration. These findings are consistent with the observations reported by Srivastava et al. (2024) and Tsikopoulos et al. (2021), reinforcing the benign nature of irritation fibromas and the absence of cellular atypia [1,3]. Such features are characteristic of irritation fibroma and correspond to the histological criteria described by Barker and Lucas [6], who defined these lesions as nodular masses of fibrous connective tissue covered by stratified squamous epithelium. The histopathological findings observed in the present case were consistent with those previously described in the literature.

The differential diagnosis of irritation fibroma includes several common oral lesions, such as pyogenic granuloma, peripheral ossifying fibroma, peripheral odontogenic fibroma, benign salivary gland neoplasms, lipoma, dermoid cyst, neurofibroma, squamous papilloma, and odontogenic tumors. Although these lesions may present with clinical features similar to irritation fibroma, they differ significantly in etiology and histopathological characteristics. Accurate differentiation is therefore essential to ensure appropriate diagnosis and treatment, as emphasized by Srivastava et al. (2024) [1].

The standard treatment for irritation fibroma is surgical excision, which is generally associated with a favorable prognosis and low recurrence rates. For smaller exophytic lesions, diode laser excision has also been proposed as an alternative treatment modality. This technique offers several advantages, including reduced postoperative pain, elimination of the need for sutures, and faster healing, as discussed by Ortega-Concepción et al. (2017) [7].

In the present case, adjunctive postoperative management strategies were used following surgical excision of the lesion. These included the use of a zinc-containing thermoplastic palatal stent in combination with topical oxygen- and lactoferrin-releasing oral gel during secondary healing. The stent was primarily used for palatal wound protection and patient comfort during the postoperative healing period.

Zinc-containing thermoplastic polymer granules have recently been introduced for chairside fabrication of palatal stents, as zinc has been shown to support wound healing by reducing postoperative pain and bleeding while accelerating re-epithelialization. A randomized controlled clinical trial by Alkaya et al. (2023) evaluated the effectiveness of chairside-fabricated zinc-containing palatal stents compared with gelatin-based hemostatic suturing following free gingival graft surgery [8]. The authors reported that zinc-containing stents significantly reduced postoperative pain and bleeding during the early healing phase, accelerated re-epithelialization, and reduced surgical time. In the present case, satisfactory postoperative healing was observed; however, due to the descriptive nature of a single case report, no direct conclusions regarding therapeutic efficacy can be established.

Adjunctive topical therapies have also been proposed to support postoperative wound care. Oxygen- and lactoferrin-releasing agents have been described in previous studies for their potential antimicrobial and anti-inflammatory properties. Oxygen delivery to the surgical site may promote wound healing by enhancing collagen synthesis, angiogenesis, and bacterial inhibition. Lactoferrin, known for its anti-inflammatory and antimicrobial properties, may also support soft tissue regeneration through stimulation of fibroblast and keratinocyte activity [9-14]. In the present case, topical oxygen- and lactoferrin-releasing gel was used as an adjunctive postoperative measure during secondary healing. However, this case report does not allow determination of the specific clinical contribution of these adjunctive therapies to the observed healing outcome.

The prognosis of irritation fibroma is generally excellent, with a very low risk of malignant transformation [1]. Nevertheless, clinicians should maintain a broad differential diagnosis, particularly in cases of atypical presentation or rapid lesion growth. This highlights the importance of comprehensive clinical and histopathological evaluation in all suspected cases. Incompletely excised lesions have been associated with recurrence rates ranging from 8% to 20%, emphasizing the importance of complete surgical removal and long-term follow-up [1-3]. As reported by Tsikopoulos et al. (2021), follow-up periods ranging from six months to one year are recommended to monitor healing and detect possible recurrence [3].

## Conclusions

This case report highlights the unusual presentation of a large palatal irritation fibroma measuring 2.7 cm in diameter, significantly exceeding the size commonly reported in the literature. Despite an initial benign diagnosis following incisional biopsy, the patient neglected further treatment, allowing the lesion to progressively enlarge over a three-year period. This emphasizes the importance of both clinician and patient awareness regarding the need for appropriate follow-up and definitive management of benign oral lesions.

Complete surgical excision resulted in satisfactory healing, with no evidence of recurrence at one-year follow-up. Postoperative management included the adjunctive use of a zinc-infused thermoplastic palatal stent combined with topical oxygen- and lactoferrin-releasing oral gel during secondary healing. Further clinical studies are required to evaluate their potential role in postoperative wound management following oral surgery.
